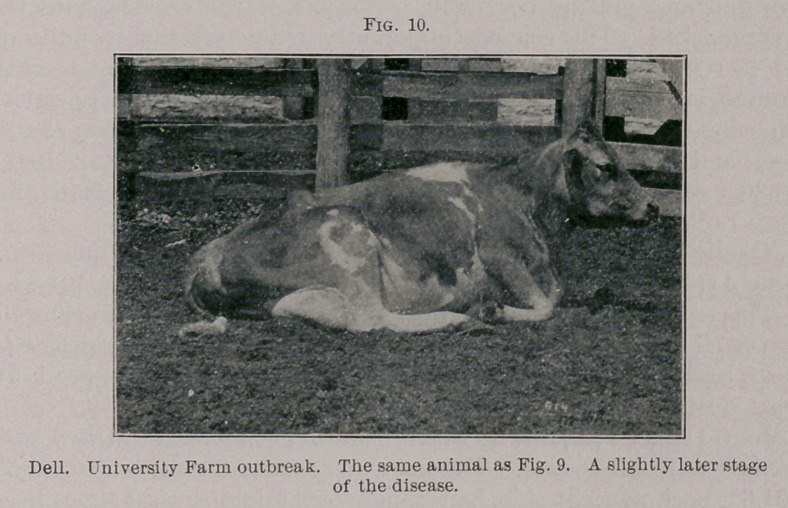# Hemorrhagic Septicæmia

**Published:** 1903-02

**Authors:** M. H. Reynolds

**Affiliations:** University of Minnesota, St. Anthony’s Park, Minn.


(To be continued.)

---

### HEMORRHAGIC SEPTICÆMIA.

By M. H. Reynolds, M.D., V.M.,
UNIVERSITY OF MINNESOTA, ST. ANTHONY'S PARK, MINN.

*Provisional Report on Bacteriological Examination of Hemorrhagic Septicæmia at State Experiment Station, St. Anthony's Park, June* 9, 1902.

(Continued from page 29.)

Specimens were collected from cows Nos. 1, 2, and 3 on June 9th, and from cow No. 4 on June 12th, at autopsies conducted by Drs. Reynolds and Brimhall. Bacillus bovisepticus (hemorrhagic septicæmia) was obtained in pure culture from the liver and spleen of cow No. 4, and was found present also in the lung and meninges of cow No. 1, and in the pharyngeal gland and meninges of cow No. 2, though in these latter two animals the bacillus was mixed with other organisms, such as colon bacillus. This was probably due to the fact that the autopsies were not made until several hours after death.

With the cultures obtained from the meninges of cow No. 1, rabbit No. 569 was inoculated intravenously June 13th and died on June 14th—*i. e.*, in less than twenty-four hours. From the rabbit the bacillus was obtained in pure culture from the heart's blood.

From cow No. 3 this bacillus was not isolated, probably owing to the very great infection with other micro-organisms which had developed after the death of the cow. Further rabbit inoculations, will be made. In the meantime, from three of the four sources the micro-organism has been obtained, and from one source, cow No. 1, the organism has been shown to be virulent. The strains of bacilli from the other two cows have been inoculated into animals, but as yet no results have been obtained.

Yours truly,
F. F. WESBROOK.

*University Farm Case Notes.* Iris. June 8th, 3 to 5 P.M., she was slightly stupid, in standing position, apparently strong and breathing easily. This cow drank naturally and did not show anything unusual except a slight listlessness. She died at 6.15 P.M. Iris' death was very unexpected until within a few minutes before it occurred. (Fig. 7.)

FIG. 7.

Iris. University Farm outbreak. An apparently easy attitude, slightly stupid. A moment before she was drinking and switching flies unconcernedly. The photograph shows her condition at 5 P.M.; at 6.15 P.M. she was dead.

Vye. June 8th, 11 A.M., quiet, apparently comfortable, could walk fairly well; not supposed to be in any serious danger. This cow had a slight convulsion at 9 A.M.; 1 to 3 P.M. quiet and lying in a comfortable position.

June 9th the cow was found dead early in the morning in a back stable, having forced her way through an intervening door, which had been closed the night before. She had evidently shown great activity before death, although she was very quiet the evening before and was not considered to be in immediate danger.

Lou. June 8th, 3 to 5 P.M., standing most of the time, walked fairly well, but seemed very weak. Died about 6.15 P.M. (See Fig. 8.)

Sweet Clover. June 8th, died about 9 P.M., after an hour or more of intense nervous and physical activity. She was champing her jaws spasmodically and had convulsions of the face and neck mus-

FIG. 8.

Lou. University Farm outbreak, meningeal type; case in first stage, cow stupid.

cles. The earlier history of this case is unknown. This heifer was taken sick suddenly in a pasture to which the other cases had not had access and was the only case to develop in this pasture.

FIG. 9.

Dell. University Farm outbreak, meningeal type; case in first stage, animal stupid.

Alzanka. June 8th, quiet at 10 to 12 A.M. Neck around to the side, as in parturient paresis. Could walk, but was down most of the time. 3 to 5 P.M., down all the time; neck in the flank; quiet.

9 P.M., down with the neck in the right flank most of the time; quiet, stupid, with stertorous breathing.

June 9th, 9 A.M., about the same as the previous night; 11 A.M., temperature 100.8°; 2.30 P.M., 101.8°; 6.45 P.M., 102.8°; 9.40 P.M., 101.8°. June 10th, 7 A.M., 100.8°. This cow died at 10 P.M. Little apparent change in condition until near the end.

Dell. June 8th, 11 A.M., quiet, down most of the time. At 3 P.M. lying in the yard, stupid, neck bent to one side. She was quiet, although the appearance of her eyes and the condition of the cervical and facial muscles suggested a tension of the nervous system. Breathing at this time was stertorous; 10 P.M., temperature 101.5°; down, quiet, but showing the usual symptoms in the face and neck; loss of skin sensation, etc.

FIG. 10.

Dell. University Farm outbreak. The same animal as Fig. 9. A slightly later stage of the disease.

June 9th, 9 A.M., apparently little change since last night; 2.30 P.M., about the same; 6.45 P.M., temperature 101.6°; 9.50 P.M., 103.4°. This cow died at about 4 A.M., June 10th.

Countess. June 8th, 11 A.M., respiration stertorous, recumbent most of the time, but could walk. 2.30 P.M., pupil of the right eye was contracted, the left dilated; 3.05 P.M., this cow was down most of the time, respiration stertorous; could walk, but the gait was quite irregular.

June 9th, 9 A.M., cow had died during the night and was found out of doors, having in some way forced her way through or under a very heavy sliding door. Evidently there had been intense activity before death.

Euroma. This was a Jersey cow, giving normally at this time about fourteen pounds of milk, testing 5 to 6 per cent. butter fat.

She gave on the evening of June 6th, 5.1 pounds of milk, testing 6.2 per cent.; on the morning of the 7th, 2.1 pounds of milk, 5.2 per cent. butter fat.

On the morning of the 7th the head was carried to the left, the left ear was more upright than the other and held back in a peculiar position, and the animal seemed stupid.

June 8th, 9 P.M., the patient was standing grating her teeth and showing very marked spasms of the cervical muscles. The head was now turned around to the right and a portion of the right ear was cold. She was not seen during the interval, but supposed to be quiet and easy from what was learned of the attendant. At 9.05 P.M. this cow was found back of a spray pump in the runway, very stupid, weak, and with poor circulation. She was in a standing position and grating her teeth. Spasms of the cervical muscles were marked. This cow was apparently in a very serious condition. At 10 P.M. there was great nervous excitement, the patient tearing around in a large room with short intervals of comparative quiet. Chewing motion, discharge of frothy saliva, and convulsions of the neck and face muscles were continuous; 10 P.M., temperature 104°; 10.30 P.M., 105°; 11.20 P.M., 107.6°; died at 11.40 P.M. Note the very rapid rise of temperature.

Trudie Lee. This cow gave no milk on the evening of June 10th. June 11th, 10 A.M., temperature 102°; 1 P.M., temperature 101.8°.

This cow was apparently almost normal on June 11th, but showed the usual peculiar expression of eyes and head. She was grating the teeth slightly, salivation was increased, and skin sensation good, at least during the forenoon; patient slightly dull.

June 11th, 1 P.M., down; when made to get up she stretched and seemed to feel first-rate. The nose was moist; 6 P.M., temperature 101.6°; neck at right side, hair rough, feet raised several times in a crampy way, nose moist; 9 P.M., temperature 102.6°, wild expression in the eyes, and nose dry. She died during the night.

*Examination Post-mortem.* Only this one autopsy record of the University Farm cases will be given here. The findings in all cases were very similar, and Trudie Lee may be taken as a type.

Trudie Lee, a Jersey cow, in good condition, died early in the morning of June 12th. The carcass was in fairly good condition. There were hemorrhages in several places on superficial parts, under scapulæ, etc. There were very marked hemorrhages involving the meninges of the medulla, but scarcely showing at all on the brain surface or in its substance. Multiple hemorrhages were thickly scattered over the omentum and mesentery, and there were several on the surface of the liver. The heart showed many small hemorrhages on the surface, the right auricle being very markedly hemorrhagic. On the costal pleura there were numerous hemorrhages of varying sizes. The lungs were deeply congested in places, especially in the region of the internal faces.

Lesions were all of marked hemorrhagic character. There were two marked hemorrhages between the peritoneal and muscular coats of the uterus, which contained a normal five or six months' fœtus.

*Comments on Case Notes.* A survey of the foregoing case notes brings to light several interesting points. In the first place, it will be noted that the temperatures were normal or subnormal rather than high, until a very short time before death, when the temperature rose very rapidly, notably in the case of Euroma. The evidence on this point is not altogether satisfactory, for in so many of the cases circumstances were such that temperatures could not be well taken during the last hour or so. In the Caffrey cases, where it was possible to follow the cases entirely through its course from the onset until the fatal termination, the temperatures were normal or subnormal throughout.

In an outbreak which occurred among cattle at the University Experiment Farm the disturbances of the nervous system were particularly marked; so much so that the diagnosis based on both ante-mortem and post-mortem symptoms was unanimously considered to be cerebro-spinal meningitis.

Several of these cases at certain stages very closely resembled typical cases of parturient paresis. (See letter from Dr. Hela, under "Caffrey Outbreak.") It should perhaps be noted that we had one cow taken sick with all the clinical symptoms of this latter disease, the symptoms appearing about thirty-six hours after parturition. (See "A Supposed Milk-fever Case.") The outbreak previously described as appearing among the dairy cattle belonging to the University Experiment Farm appeared on June 8th, or about eleven days after this supposed milk-fever case. No examination post-mortem was made of the latter, but in view of the fact that several of the cases which appeared in the general outbreak among our cattle very closely resembled milk fever in all points except in the history of recent parturition, grave doubt has arisen in the mind of the writer whether the supposed milk-fever case was not a cerebro-spinal type of hemorrhagic septicæmia instead of parturient paresis. The writer can well understand that a suspicion as to the accuracy of the diagnosis in an apparently typical case of parturient paresis may appear somewhat peculiar, to say the least, but to those of us who saw the cases among our University Farm cattle it does not seem peculiar at all. There does not appear any reason why this peculiar type of hemorrhagic septicæmia could not appear thirty-six hours after parturition as well as at any other period. If we consider the sudden checking of milk flow, the constipation, the posterior paralysis, the lack of skin sensation, the recumbent position, with the head in the flank much of the time, the reason for doubting an apparently clear diagnosis may be easily understood.

A peculiar fact which appeared in connection with our University Farm cases was noticed by the attendants, and everyone who saw the case, viz., that the animals nearly all died in what they called the "inverse ratio"—*i. e.*, the cases which were apparently most seriously sick in their histories were the cases which lived the longest, whereas the apparently milder cases died very quickly and very unexpectedly. Those cases which were apparently most seriously sick were the ones which lived until the last ones of the outbreak. The Vye cow is an instance in point. If the brief convulsions on the morning of June 8th had not been seen, this cow would ordinarily not have been considered sick at all beyond a very slight diarrhœa.

Iris was standing in the yard drinking, switching flies, showing nothing whatever apparently wrong with her except slight listlessness, as seen in the accompanying photograph, and yet she died very suddenly and unexpectedly, without developing serious symptoms until a very short time before death.

In none of the cases witnessed by the writer has there been a rise of temperature, not any tenderness on pressure over the spinal column more than elsewhere over the body, but quite a number have shown a hypersensitive condition of the skin in general. None of the cases seen by the writer presented unnatural heat at the base of the horns, or throat paralysis. It will be seen that although the State Farm outbreak was unquestionably a cerebro-spinal meningitis, as proven by ante-mortem and post-mortem symptoms, yet it differed in very many particulars from cerebro-spinal meningitis as it appears in the human family.

*A Case of Ovine Hemorrhagic Septicæmia.* A show sheep in fine condition, ten months old, which arrived from Canada about the middle of February, was noticed sick February 17th and died on the 18th. This animal was examined on the 17th at about 4 P.M. The patient showed peculiar breathing, there being two or three short, moderately full respirations, and then a considerable interval. Respirations were not very rapid, and quite unlike an ordinary pneumonia. Neither nasal discharge nor cough was noticed; temperature and pulse were not taken. This was supposed to be a case of common catarrh and not thought serious. The animal died very unexpectedly.

*Autopsy.* Several bright, sharply-defined, hemorrhagic areas were found on the inner surface of the skin after removing an excessive amount of fat. There were no hemorrhages on the superficial muscles. The small intestines were evenly and generally congested, but this was comparatively slight. No hemorrhage upon any portion of the alimentary tract. The lungs were as if they had been taken from a hog during an outbreak of hog cholera and swine-plague, and were typical of the latter disease. The collapsed areas amounted to probably one-third of the entire lung substance. The heart showed extensive hemorrhagic infiltrations, especially the auricles. Report from Dr. Wesbrook, Director of the Bacteriological Laboratory of the State Board of Health, was to the effect that pure cultures of bacillus bovisepticus were recovered.

*Supposed Milk-fever Case.* A Jersey cow, of high dairy type, belonging to the University Experimental Farm, calved May 28. She was noticed sick on May 29th, and when seen by the writer had lost voluntary control of the limbs; skin sensation was poor over most of the body surface. She was rather quiet, with the head in the flank, and the usual retention of feces and urine. Iodide of potassium, ten grammes, was dissolved in a quart of warm water, at 10.30 A.M., May 29th. One-fourth of this was injected into each gland. This treatment apparently had very little effect and the dose was repeated at 9 A.M., May 30th. This second dose was practically without effect, and the cow died some time during the same afternoon. We did not expect the cow to die at this time, and she was not seen during the last few hours. In view of the symptoms which were seen in one general outbreak of hemorrhagic septicæmia where several cases very closely simulated milk fever, the suggestion may not appear unreasonable that this case was either not a case of milk fever, or else, if you please, a case of milk fever caused by the same germ which was apparently responsible for the development of the other cases, which appeared later. (See University Farm Outbreak.)

## Figures and Tables

**Fig. 7. f1:**
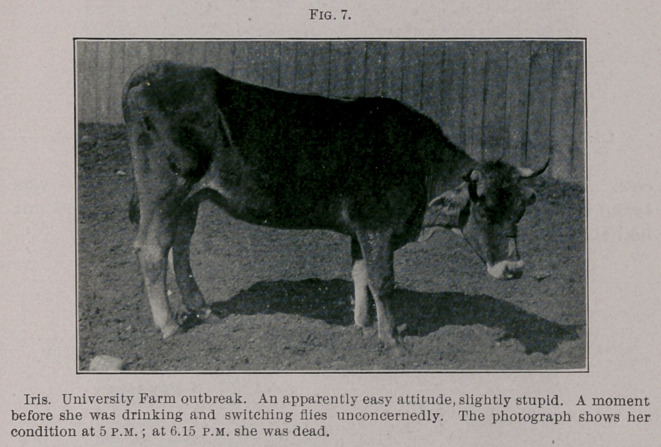


**Fig. 8. f2:**
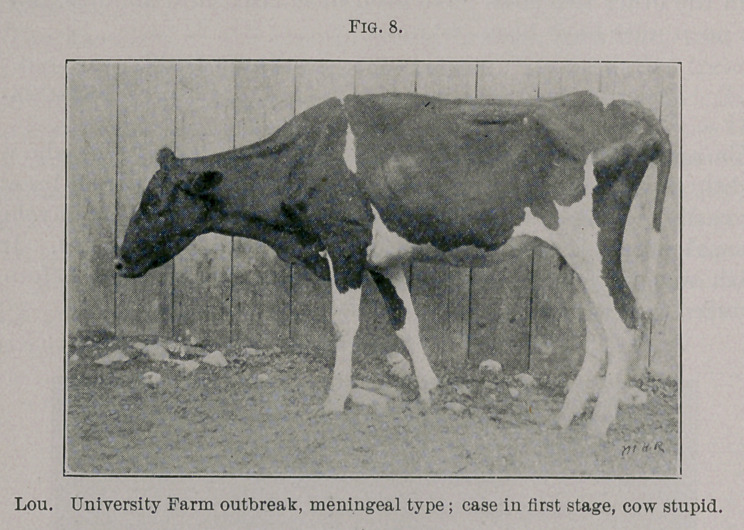


**Fig. 9. f3:**
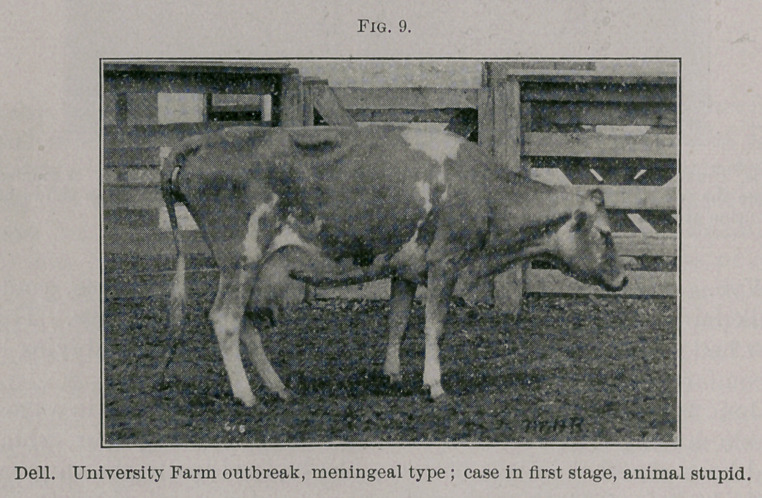


**Fig. 10. f4:**